# Seasonal diet partition among top predators of a small island, Iriomote Island in the Ryukyu Archipelago, Japan

**DOI:** 10.1038/s41598-024-58204-6

**Published:** 2024-04-02

**Authors:** Alisa Tobe, Yukuto Sato, Nakatada Wachi, Nozomi Nakanishi, Masako Izawa

**Affiliations:** 1 Faculty of Science, University of the Ryukyus, Nishihara, Okinawa Japan; 2 Center for Strategic Research Project, Organization for Research Promotion, University of the Ryukyus, Nishihara, Okinawa Japan; 3 Present Address: Wildlife Research Center, Kyoto University, Sakyo, Kyoto Japan; 4 Present Address: Research Laboratory Center, Faculty of Medicine, University of the Ryukyus, Nishihara, Okinawa Japan; 5 Present Address: Iriomote Station, Tropical Biosphere Research Center, University of the Ryukyus, Taketomi, Okinawa Japan; 6 Present Address: Kitakyushu Museum of Natural History and Human History, Kitakyushu, Fukuoka Japan

**Keywords:** Ecological networks, Ecosystem ecology, Molecular ecology

## Abstract

Small islands tend to lack predators because species at higher trophic levels often cannot survive. However, two exceptional top predators—the Iriomote cat *Prionailurus bengalensis iriomotensis*, and the Crested Serpent Eagle *Spilornis cheela perplexus*—live on the small Iriomote Island in the Ryukyu Archipelago. To understand how these predators coexist with limited resources, we focused on their seasonal diets between which conflicts are considered to occur. To compare the diets, we used DNA metabarcoding analysis of faecal samples. In the summer, we identified 16 unique prey items from Iriomote cat faecal samples and 15 unique prey items from Crested Serpent Eagle faecal samples. In the winter, we identified 37 and 14, respectively. Using a non-metric multidimensional scaling and a permutational multivariate analysis of variance, our study reveals significant differences in the diet composition at the order level between the predators during both seasons. Furthermore, although some prey items at the species-to-order level overlapped between them, the frequency of occurrence of most prey items differed in both seasons. These results suggest that this difference in diets is one of the reasons why the Iriomote cat and the Crested Serpent Eagle are able to coexist on such a small island.

## Introduction

Island ecosystems exhibit unique ecological characteristics that are sometimes simpler or more monotonic than those of continental ecosystems. This is mainly due to a limited number of species and individuals, with immigration and emigration events less likely to occur^1,2^. On such islands, predators at higher trophic levels are often absent, because predator species, such as carnivores, cannot maintain their populations without sufficient prey resources^3^.

The Iriomote cat (IRC) *Prionailurus bengalensis iriomotensis* is a top predator on Iriomote Island, located in the southern part of the Ryukyu Archipelago, which covers an area of only approximately 289 km^2^^4^. The leopard cat *P. bengalensis* is widely distributed in East and Southeast Asia, and IRC is a subspecies endemic to Iriomote island^5^. The food habits of IRC are already well studied^6–10^. Notably, IRC feed on a variety of prey animals, such as mammals, birds, reptiles, amphibians, fish, insects, and crustaceans depending on seasonal prey availability^9^, this contrasts leopard cats in other regions who primarily feed on rodents^11–14^. While Iriomote Island has no native insectivore or rodent species unlike other regions inhabited by leopard cats^15^, the biomass of frogs is extremely high^16^. Frogs are amongst the most detected species in diet analyses of IRC^6,9^. Previous study concluded that this seasonally plastic food habit and the rich biomass of frogs enable IRC to live on such a small island^9^. However, these studies did not consider competition with other predator species.

The Crested Serpent Eagle (CSE) *Spilornis cheela perplexus* is another residential top predator of Iriomote Island. The CSE is endemic to Iriomote and Ishigaki Island in the Ryukyu Archipelago and is a subspecies of the Crested Serpent Eagle *S. cheela*, which is widely distributed in East and South Asia^17^. CSE on Iriomote Island shows carnivorous food habit and feeds on a variety of prey animals, much like the IRC^18,19^, most of which overlap between IRC and CSE. Indeed, CSE on Iriomote Island is shown to prey on a more varied number of species compared to CSE in other regions who mostly prey on snakes^20–22^. These similarities may give rise to conflict for food resources between the IRC and CSE^23^.

Detailed comparison of the diets of IRC and CSE based on previous studies is difficult because the analysis methodologies used have been different between the two species. Previous diet analysis of IRC has been based on morphological observations of faecal samples and the stomach contents of dead individuals^6–10^. Analysis of CSE faecal content is more challenging, as carnivorous birds often spit out undigested items such as pellets, and only soluble materials are excreted as faeces. Therefore, previous studies on the CSE diet have largely been limited to the stomach content of dead individuals and direct observation of feeding behaviors^18,19^. The difficulty in obtaining dead individuals and the limited frequency of direct observations led previous studies to have insufficient sample sizes.

The distribution of other subspecies of leopard cats and Crested Serpent Eagles is mostly overlapping^5,17^. Because of the lack of the ecological studies of Crested Serpent Eagle, diet information of sympatric species is missing. Iriomote Island is the smallest island where leopard cats and Crested Serpent Eagles coexist and the food habits of both of them are wider than each subspecies. Although some studies have compared the diets of sympatric animals that exhibit similar ecological niches^22,24–26^, few studies have focused on sympatric predators endemic to small islands such as Iriomote Island. The mechanisms of coexistence of these predators are important factors for understanding the ecological features of a small island with its limited resources.

**Figure 1:**
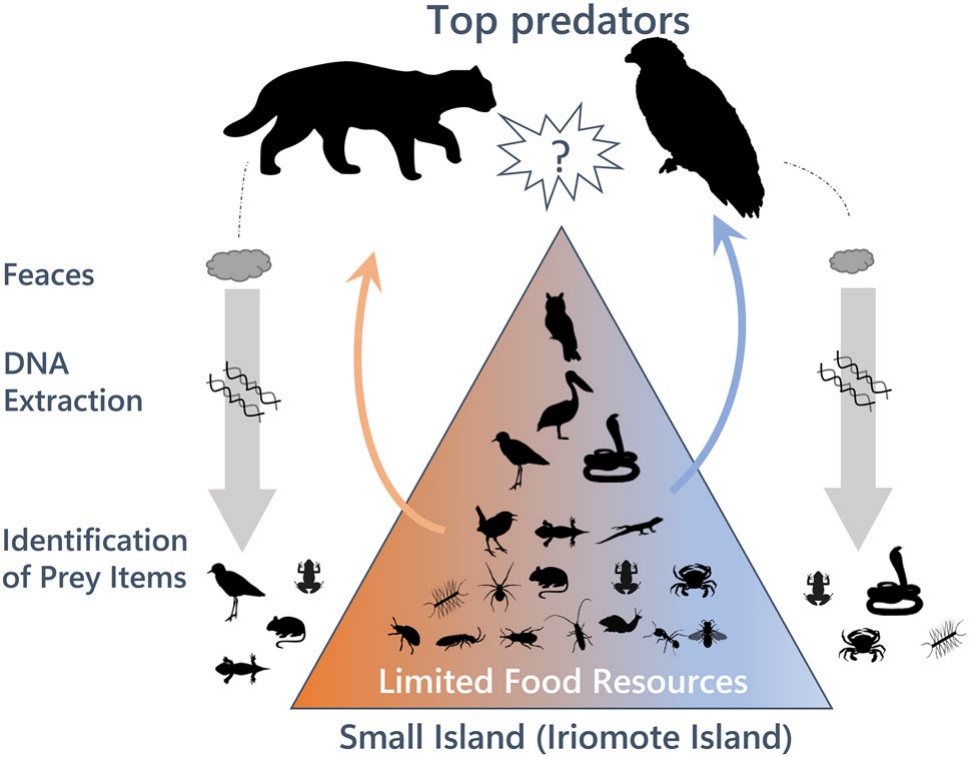
The outline of the present study. Silhouettes were gained by phylopic (https://beta.phylopic.org/).

We performed DNA barcoding analysis of the faeces of both IRC and CSE to examine their prey species. DNA barcoding generally provides a higher taxonomic resolution than morphological observations of faecal or stomach samples^27,28^. It has relatively recently gained moment as a major method for animal diet analysis. This method enabled us to analyze CSE diets using faecal samples with larger sample sizes than stomach content analysis or direct observations. Previously, studies in Pakistan and China used DNA barcoding to investigate the diets of other subspecies of the leopard cat^29–32^. However, no studies have addressed any CSE species. DNA barcoding allowed us to conduct a direct comparison analysis of the prey of a bird and mammal species without necessitating the utilization of different methods. Our goal with conducting this analysis was to explicitly examine the competition between IRC and CSE for limited food resources on a small island using the same and higher resolution method for both species (Fig. 1).

## Results

### Faecal sampling and DNA sequencing results

**Figure 2:**
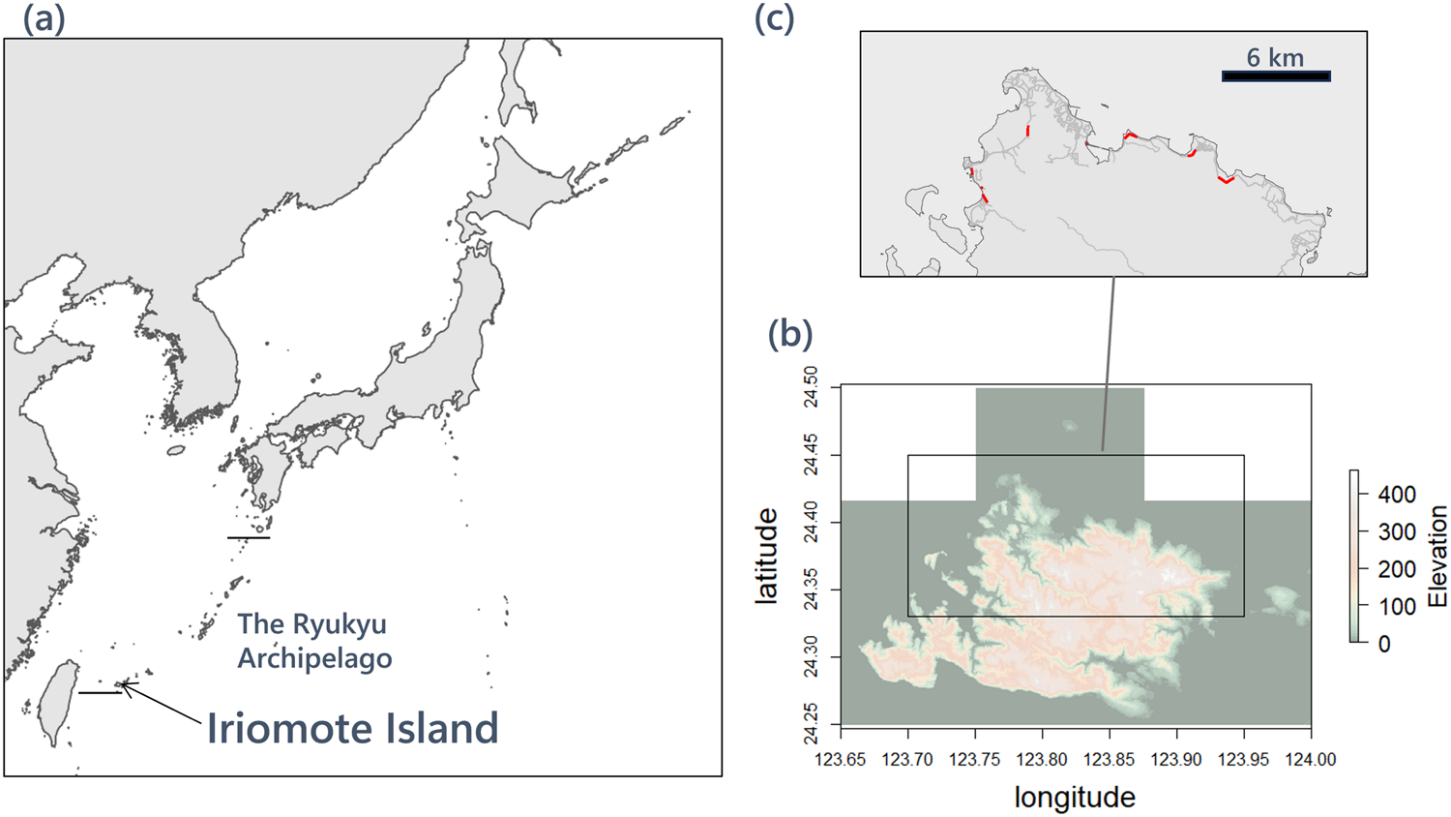
(**a**) A map showing the location of Iriomote Island. (**b**) A map of Iriomote Island and study area. (**c**) The eight transect lines (marked in red) used in this study. All maps were made in R ver. 4.3.0 based on information from the Technical Report of the Geospatial Information Authority of Japan https://www.gsi.go.jp/.

Faecal samples were collected from transect lines that we set on Iriomote Island (Fig. 2) and also provided by Okinawa District Forest Office of Forest Agency and Ministry of the Environment of Japan and Iriomote Wildlife Conservation Center of the Ministry of the Environment of Japan. From IRC, we obtained 38 and 76 faecal samples in the summer and winter, respectively. For CSE, we collected 24 and 12faecal samples in the summer and winter, respectively. The number of IRC samples that contained DNA sequences from more than one prey item was 31 (81.6%) in summer and 64 (84.2%) in winter. For CSE, 21 samples (87.5%) during summer and nine samples (75.0%) during winter contained DNA sequences from more than one prey item. These samples were used for subsequent analyses. Using DNA metabarcoding sequencing results, we obtained 333 sequencing reads using the COI#1; 10,335 sequencing reads using the COI#2; 208,673 sequencing reads using the Ecoprimer; and 74,483 sequencing reads using the MtAnr. These sequences were derived from 55 different prey items, including five species of Mammalia; 17 species, one genus, one order, and one family of Aves; ten species and one genus of Reptilia; seven species and one family of Amphibia; one species of Osteichthyes; three species of Malacostraca; four species and one order of Insecta; and two families of Chilopoda (Supplementary Table S2). We could identify 83.5% of the MOTUs at the species level.

### Characteristics of detected prey species by each primer set

**Figure 3:**
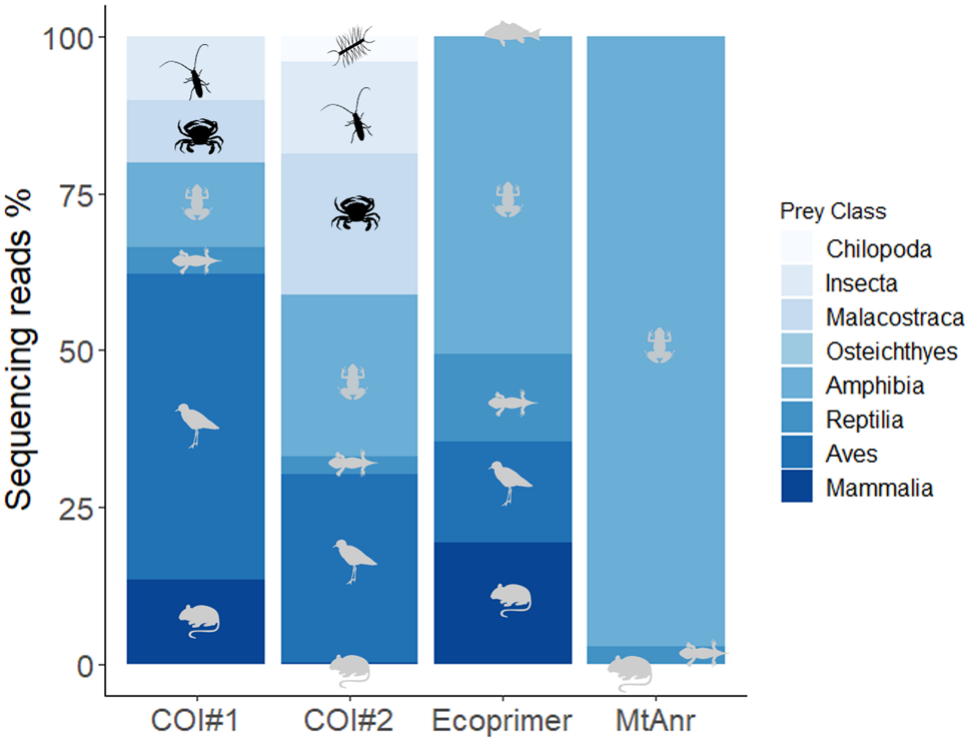
Percentage of sequencing reads of detected prey class in each universal primer set. Silhouettes shown in light grey are vertebrates, shown in black are invertebrates. Silhouettes were gained by phylopic (https://beta.phylopic.org/).

The number of sequencing reads for the detected prey taxa varied remarkably among primer sets. Invertebrates were detected using COI#1 and COI#2, whereas vertebrates were detected using all primer sets (Fig. 3). The COI#1 detected the Brown-eared Bulbul *Hypsipetes amaurotis stejnegeri* which was not detected by any other primer sets. COI#2 detected a relatively large number of invertebrates, such as the mudflat crab *Chiromantes dehaani*, the blue land crab *Discoplax hirtipes*, the vine hawkmoth *Hippotion celerio*, the bush cricket *Mecopoda elongate*, the privet hawkmoth *Psilogramma menephron*, and centipedes *Scolopendromorpha* sp*.*, that were not detected by any other primer sets. The Ecoprimer detected each class of land vertebrates almost evenly. In particular, Osteichthyes (the barred mudskipper *Periophthalmus argentilineatus*) was not detected by any other primer sets, and was only detected in one faecal sample (Fig. 3). The sequencing reads derived from Amphibia accounted for 97.2% of the total number of sequencing reads obtained by the MtAnr. The Yaeyama kajika frog *Buergeria choui* and Utsunomiya’s tip-nosed frog *Odorrana utsunomiyaorum* were detected only using this primer set.

### The estimated diet composition of IRC and CSE

**Figure 4:**
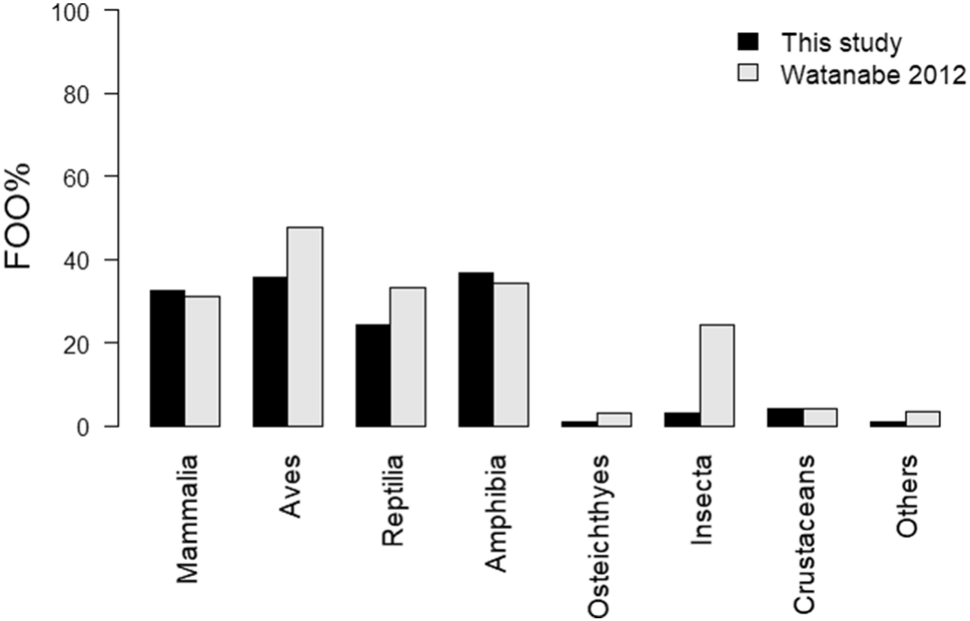
Comparison of frequency of occurrence (FOO) of each IRC prey class between this study (*N* = 95) and previous study that used morphological observation of faecal contents (*N* = 947)^9^.

The number of prey items detected from the faecal samples of each predator during each season was as follows—summer samples of IRC: 16 items belonging to 14 species, one genus, and one order belonging to five classes, nine orders, 12 families, and 15 genera; summer samples of CSE: 15 items belonging to 12 species, one genus, and two families belonging to five classes, five orders, 11 families, and 12 genera; winter samples of IRC: 37 items belonging to 30 species, two genera, three families, and two orders belonging to eight classes, 16 orders, 23 families, and 30 genera; winter samples of CSE: 14 items belonging to 12 species and two families belonging to six classes, seven orders, 12 families, and 12 genera (Fig. 7, Supplementary Table S2). The prey taxonomic richness for each predator showed no significant difference between the seasons (Supplementary Fig. S1). Based on a comparison of the FOO values of IRC prey items obtained in this study with those obtained in a previous study^9^, except for Aves and Insecta, there were no significant differences in the FOO values of Mammalia, Reptilia, Amphibia, and Osteichthyes (Fig. 4). It should be noted that Aves and Insecta were significantly less frequent in this study than in the previous study (Fig. 4; *p* < 0.05 and *p* < 0.01, respectively).

**Figure 5:**
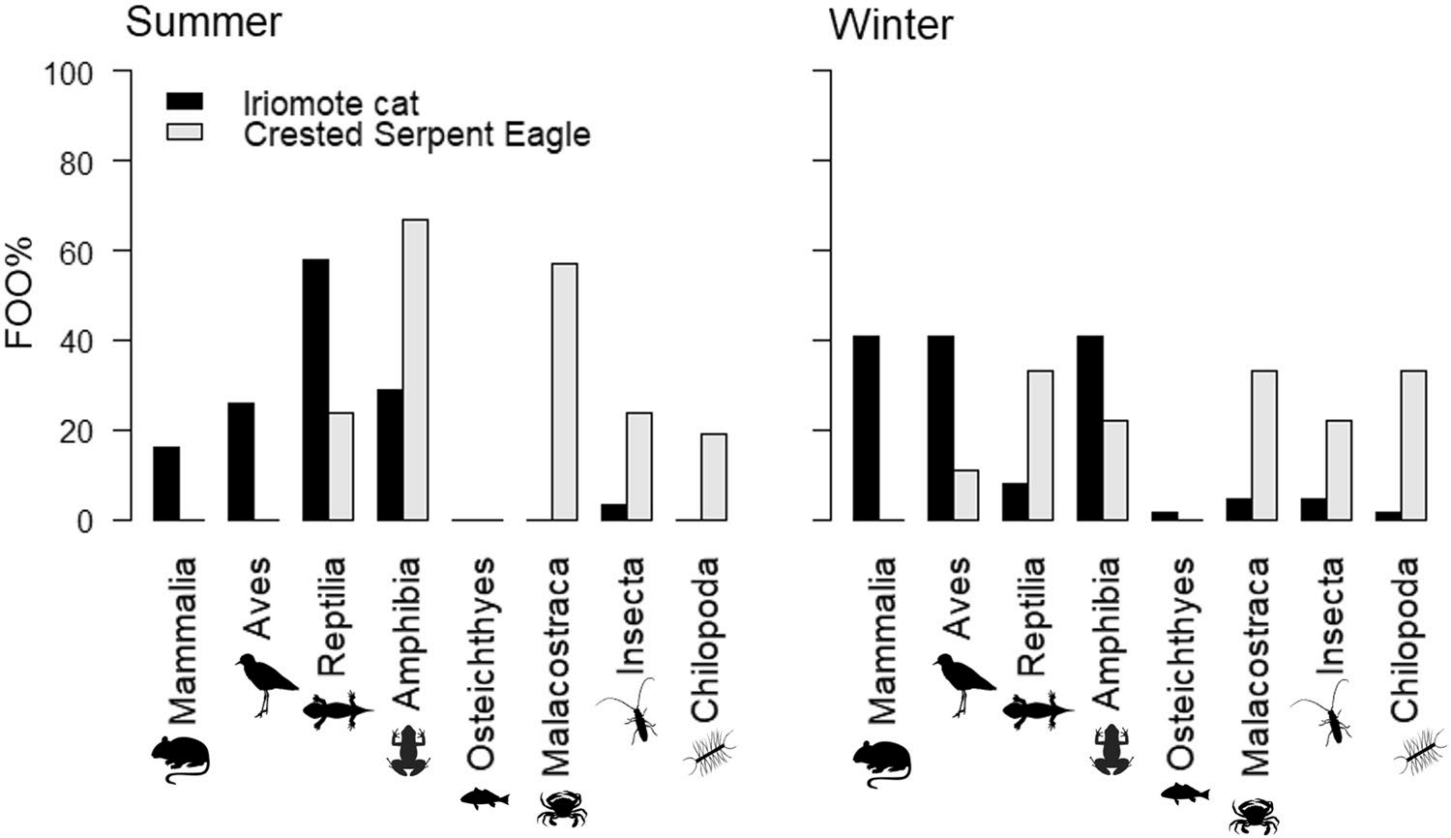
Frequency of occurrence (FOO) of each prey class detected in the IRC and CSE in each season. Silhouettes were gained by phylopic (https://beta.phylopic.org/).

Regarding the detected prey classes, Mammalia was not detected in any CSE samples, while they were frequently detected in IRC samples (Fig. 5). The FOO values of the Mammalia were significantly different between the two predators in winter (*p* < 0.05). In contrast, Malacostraca and Chilopoda were rarely detected in IRC samples while they were frequently detected in CSE samples (Fig. 5). These difference between CSE and IRC was significant in both seasons (*p* < 0.01 in Malacostraca in summer and Chilopoda in winter, *p* < 0.05 in Chilopoda in summer and Malacostraca in winter). Reptilia were significantly more frequent in IRC samples than in CSE samples in summer (*p* < 0.05), whereas the difference was not statistically significant in winter. On the other hand, Amphibia were significantly more frequent in CSE samples than in IRC samples in summer (*p* < 0.05), whereas this trend was not statistically significant in winter. Within each predator, the FOO of Mammalia and Reptilia detected in IRC faeces showed significant differences between seasons (*p* < 0.05, *p* < 0.01 in Mammalia and Reptilia, respectively). In addition, the FOO of Amphibia detected in CSE showed a significant difference between seasons (*p* < 0.05).

**Figure 6:**
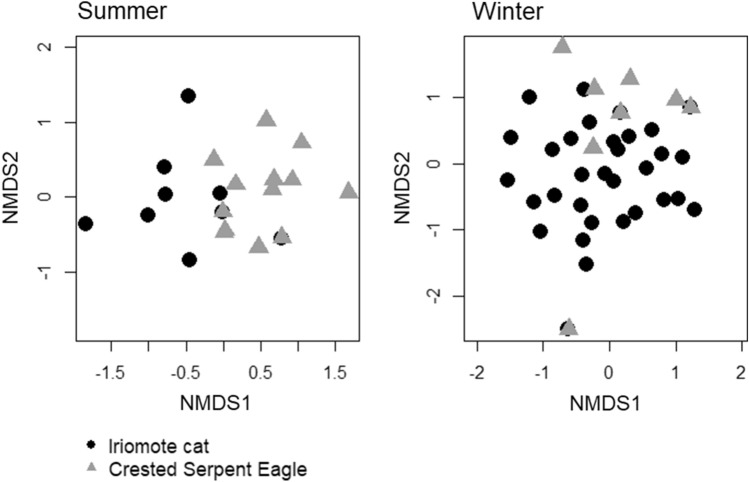
Difference in prey order composition between the IRC and CSE samples in each season. We excluded three IRC samples from summer samples because they were outliers. The stress values of NMDS are 0.041 and 0.048 respectively.

**Figure 7:**
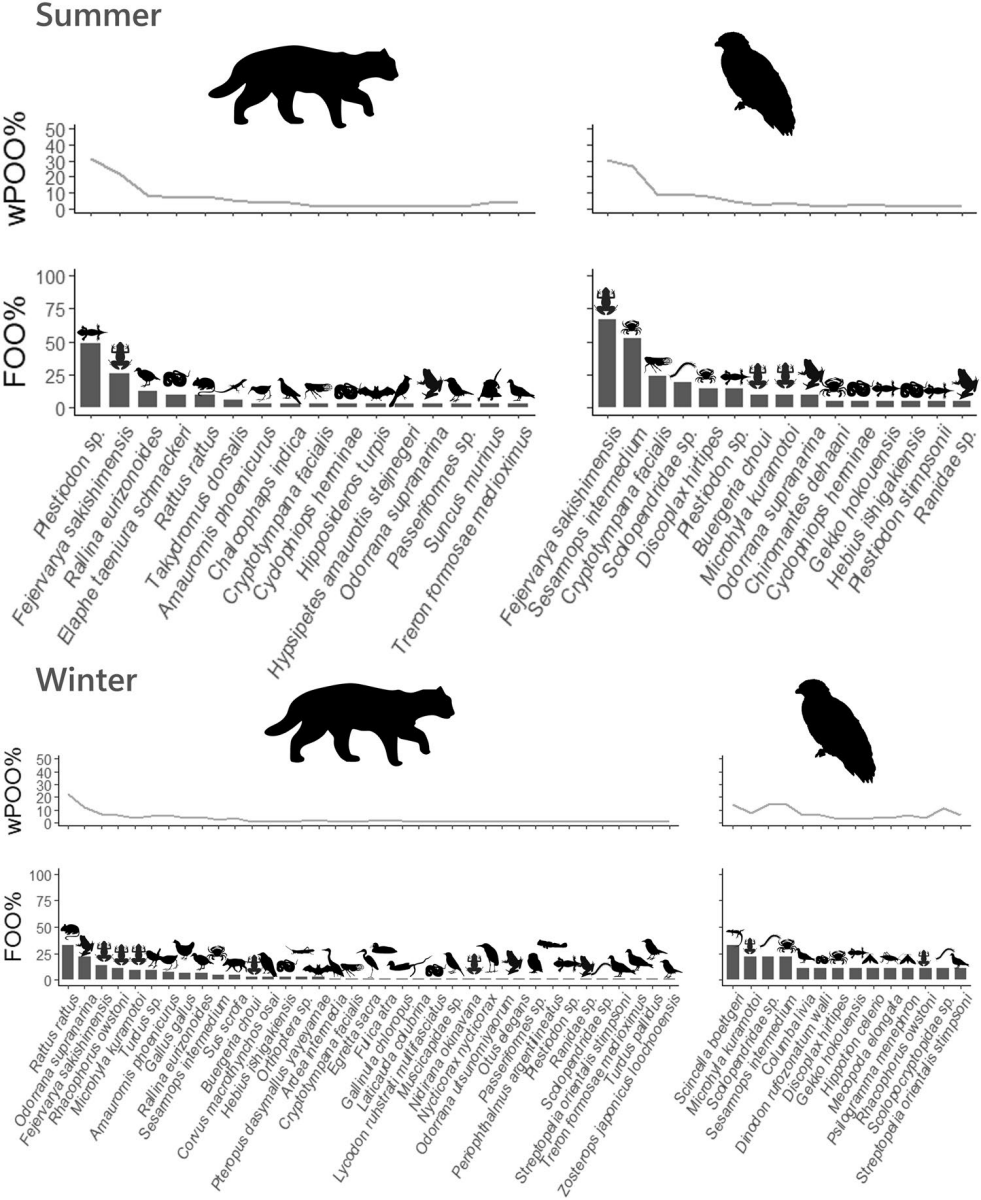
Frequency of occurrence (FOO) and weighted per cent of occurrence (wPOO) values of each prey item detected in the IRC and CSE samples in each season. In summer, 16 items were identified from IRC faeces and 15 items from CSE faeces. In winter, 37 items were identified from IRC faeces and 14 items from CSE faeces. Silhouettes were gained by phylopic (https://beta.phylopic.org/).

The NMDS and PERMANOVA analyses for comparison of diet composition between IRC and CSE showed that the prey composition at the order level was significantly different between them in both seasons (Fig. 6; *p* < 0.001) and the differences were not caused by different degrees of variation among the samples of IRC and CSE (*p* = 0.4899 in summer; *p* = 0.7077 in winter). When prey species at the species-to-order level were sorted by FOO and wPOO values, there was little difference in the ranking of prey species, with the exception of winter CSE, which had a small sample size (Fig. 7).

In Mammalia, the black rat *Rattus rattus*, had the highest FOO and wPOO values in the IRC samples in winter (Fig. 7), although FOO was not significantly higher in the IRC samples than in the CSE samples (Supplementary Table S2). In both seasons, black rats were the most frequently detected mammals in the IRC samples (Fig. 7), and their FOO was significantly higher in winter than in summer (Table S2; *P* < 0.05).

In Reptilia, the FOO and wPOO values of Japanese skinks *Plestiodon* sp., were the highest in the detected IRC prey items in summer (Fig. 7), and its FOO was significantly higher in IRC samples than in CSE samples (Supplementary Table S2; *p* < 0.05). Within the IRC samples, the FOO value of Japanese skinks was significantly higher in summer than in winter (Supplementary Table S2; *p* < 0.01). Similarly, the Sakishima beauty snake *Elaphe taeniura schmackeri*, showed a significantly higher FOO in summer than in winter in the IRC samples (Supplementary Table S2; *p* < 0.05). The detection of Sakishima smooth skink *Scincella boettgeri* was significantly more frequent in the CSE samples than in the IRC samples in winter (Supplementary Table S2; *p* < 0.01), and was significantly more frequent in winter than in summer within the CSE samples (Supplementary Table S2*p* < 0.05).

In Amphibia, the Sakishima rice frog *Fejervarya sakishimensis*, had the highest FOO and wPOO values in the detected CSE prey items in summer (Fig. 7). The FOO value of this frog was significantly higher in CSE samples than in IRC samples (Supplementary Table S2; *P* < 0.01), although this frog also had the second highest FOO and wPOO values in the detected IRC prey items during this season (Fig. 7, Supplementary Table S2). On the other hand, in winter, this frog was not detected in CSE samples, and was detected in IRC samples at a lower frequency (Fig. 7), exhibiting no significant difference between IRC and CSE in the Sakishima rice frog in winter (Supplementary Table S2). Within the CSE, the FOO of this frog was significantly higher in summer than in winter (Supplementary Table S2; *P* < 0.01). In the IRC samples in winter, the greater tip-nosed frog *Odorrana supranarina*, Sakishima rice frog *F. sakishimensis*, Owston’s green tree frog *Rhacophorus owstoni*, and Yaeyama Narrow-mouthed toad *Microhyla kuramotoi* were the second- to fifth-ranking prey in terms of the FOO value (Fig. 7). Within these Anura species, Owston’s green tree frog and Yaeyama Narrow-mouthed toad were detected also in CSE samples in winter (Fig. 7, Supplementary Table S2).

Among invertebrates, only crabs Decapoda sp., were detected as Malacostraca and only centipedes Scolopendromorpha sp., were detected as Chilopoda in the CSE samples (Supplementary Table S2). The FOO value of the black cicada *Cryptotympana facialis* was significantly higher in CSE samples than in IRC samples in summer (Supplementary Table S2; *p* < 0.05).

## Discussion

Nakanishi and Izawa^6^ utilized visual analysis of stomach contents of IRC, which is thought to provide higher resolution of prey identification than that of faecal contents^33^, and identified approximately 61.6% of prey items at the species level. The present faecal DNA metabarcoding analysis identified 85.5% of prey items at the species level (Supplementary Table S2). DNA-based methods appear to be well-suited for conducting detailed dietary analyses.

In terms of Mammalia, Reptilia, Amphibia, and Osteichthyes, DNA metabarcoding demonstrates the same level of detectability as visual analysis of faecal contents at the class level when identifying IRC prey items (Fig. 4). However, Aves and Insecta were detected less frequently in this study for several reasons. According to Watanabe^9^, winter birds that visit Iriomote Island from autumn through winter, account for 40% of bird species detected in faecal samples of IRC. In contrast, this study identified only 17.6% of the preyed upon bird species as winter birds. It is important to note that the number of migratory birds arriving in a particular area can vary significantly from year to year owing to various environmental changes^34,35^. Therefore, the annual variation in the number of winter bird visits between the samples analyzed in this study and those used by Watanabe^9^ may have influenced the results.

Insecta may not have been detected adequately using our method. One possible explanation for this is the insufficient accumulation of reference sequencing data in NCBI^36^. In particular, the amount of available sequence data from insects on Iriomote Island remains poor. Sufficient reference databases are important to use the COI region as a barcode region due to the high genetic variation in genes that encode proteins in the COI region^37,38^. Indeed, many cockroaches *Rhabdoblatta* sp*.* were identified by Watanabe^9^, whereas they were not detected in the present study. The absence of sequence information for *Rhabdoblatta* sp. from Iriomote Island leads to the possibility that ambiguity in taxonomic assignment, or the failure to amplify its sequence due to mutations in primer binding regions, contributed to the non-detection of *Rhabdoblatta* sp. Bookwalter et al.^39^ whom used a primer set amplifying the same region as the current study also noted the presence of uncertain taxonomic assignment resulting from an insufficient insect database. Collecting sequence information on insects from Iriomote Island is a necessary future step.

We will not discuss details regarding Aves and Insecta below since the reasons for the limited detection of these taxa remain unclear. Nevertheless, it is noteworthy that a significant comparison of diet between IRC and CSE was achieved in this study using the same methods for both species.

Additionally, the ranking of CSE prey frequency in winter was not accurately determined in this study due to the difference in prey species ranking by FOO and wPOO values. The inconsistency in rankings may be the result of the insufficient sample size of CSE faeces in winter. However, the comparison of the frequency of each prey species between predators and seasons is meaningful.

To consider how prey animals differed between the two predators, we focused on each prey taxon. Mammalian prey was detected in both seasons in IRC only. Malacostraca (crabs) and Chilopoda (centipedes) were detected with higher FOO in CSE compared to IRC faeces (Fig. 5). Previous studies have shown that Mammalia and crabs are one of the main prey animals for IRC and CSE, respectively, with FOO values of 30.9% in the visual analysis of IRC faecal contents and 57.1% in the analysis of CSE stomach contents^9,19^. While predation on centipedes by CSE has been observed visually^18^, it was not detected in their stomach contents presumably due to digestion^19^. Furthermore, although IRC have been shown to prey on Malacostraca and Chilopoda in the visual analysis of their faecal contents, FOO values were low with 4.22% and 0.11%, respectively^8^. Previous studies also reported that the black rat could be a target of CSE predation^18^. However, the predation frequency of the black rat by CSE remains undetermined, and was not detected in the present study. In summary, it appears that IRC feeds more frequently on Mammalia and CSE feeds more frequently on crabs and centipedes. As the FOO values of Mammalia were not significantly different between the two predators in the summer season, additional research is required to examine the predation of Mammalia by CSE, especially the black rat.

Differences in the prey animals of IRC and CSE may be attributed to their distinct feeding behaviors. IRC actively hunts animals walking on the ground^7,10^, whereas CSE exhibits a sit-and-wait, or passive, foraging strategy based on perching and searching for prey animals on the ground^18,40^. The latter passive strategy may make it difficult to capture swiftly moving, larger-sized animals such as mammals which rarely appear in open areas. The frequent detection of crabs and centipedes in CSE faeces may be explained by the fact that crabs and centipedes appear more frequently in open areas where CSE tend to hunt and exhibit slower movements.

The detection of Reptilia was significantly more frequent in IRC samples than in CSE samples during the summer. In particular, Japanese skink *Pleistodon* sp. exhibited the highest FOO and wPOO values among the detected IRC prey items, and the FOO was significantly higher than in the CSE samples (Fig. 7 and Supplementary Table S2). This is concordant with that of a previous study that identified skink as a major prey for the IRC in summer^9^. In winter, the FOO of Reptilia was not significantly different between the two predators, probably due to the decreased FOO of Sakishima beauty snakes as well as skinks in the IRC samples during this season (Fig. 7 and Supplementary Table S2). In winter, the poikilothermic reptiles exhibit decreased activity owing to lower temperatures and appear only during warm daytime periods. Thus, the nocturnal predator IRC would have fewer opportunities to feed on reptiles^9^. On the other hand, CSE did not significantly affect the FOO of Reptilia between the two seasons. It is plausible that diurnal CSE has a sufficient chance of finding skinks in both seasons. Additionally, the detection of Sakishima smooth skink was significantly more frequent in the CSE in winter than in summer, and was not observed in the IRC (Fig. 7 and Supplementary Table S2). The potential for frequent feeding on Sakishima smooth skinks in CSE is the first finding of this study and requires further investigation.

IRC and CSE samples exhibited relatively high FOO and wPOO values for Amphibia in both seasons (Fig. 5). Sakishima rice frogs were detected more frequently in the summer for both predators (Fig. 7 and Supplementary Table S2). This is possible because the biomass of the Sakishima rice frog is the highest on Iriomote Island, and its activity increases during the summer^9,16^. In CSE samples, the FOO and wPOO of this frog were the highest among all prey species in summer (Fig. 7), although frogs are generally nocturnal animals, and the FOO was significantly higher than that of the IRC samples. This may be due to the relative increase in daytime activity of the Sakishima rice frog during summer. In winter, the FOO of Sakishima rice frogs decreased significantly in the CSE samples, and the difference between the two predators was not significant. This may be related to the decreased activity of Sakishima rice frogs during winter^9^. Other amphibian prey species, the Yaeyama narrow-mouthed toad and the Owston’s green tree frog, were detected for both predators in winter (Fig. 7 and Supplementary Table S2). The supposed competition for the frogs between IRC and CSE in winter requires further discussion, particularly with a larger CSE sample size.

Diel timing may provide some elucidation to the differences in IRC and CSE activity, and their prey species. However, IRC frequently prey on diurnal birds which are considered to be resting at nighttime according to the present study as well as previous studies^6–10^. CSE do not prey on these birds as frequently as IRC^18,19^. On the other hand, CSE prey on nocturnal crabs more frequently than IRC^6–10,18,19^. This suggests that their diet is determined by interactions between not only the temporal activity patterns, but also the foraging strategies and other functions previously mentioned.

Reptilia and Amphibia were suggested to be strongly related to the competition between IRC and CSE. We detected 27.2% of the potential prey Reptilia species from each IRC and CSE (Supplementary Table S2). Along with previous studies about their diet, 63.6% of the potential prey Reptilia species have been detected in IRC and 50% in CSE^6–10,18,19^. Additionally, we detected 100% of the potential prey Amphibia from IRC and 75% from CSE. Previous studies did not detect any other prey Amphibia. We can also see from the accumulation and extrapolation curves (Supplementary Fig. S1), that there is potential for detecting more prey animals by adding more samples. However, especially for IRC, all prey species detected with relatively high FOO in previous studies were also detected in the present study with relatively high FOO. This suggests that the species which the present study did not detect are rare prey animals for IRC.

In summary, despite previous studies indicating an overlap in prey items between IRC and CSE^6–10,18,19^, we found differences in diets between IRC and CSE in winter and summer. We performed faecal DNA barcoding analysis of each predator and calculated the frequency of detection of each prey item as an indicator. This enables a comparison between IRC and CSE diets for the first time. The proposed differential feeding habits of the two predators could be attributed to fundamental ecological differences such as activity patterns, foraging strategies, and seasonal variations in the activity of prey animals. It is noteworthy that prey animals with high biomass were shared by both IRC and CSE. However, we found at least partially significant differences in the FOO of each prey animal between predators. The quantitatively different diets of IRC and CSE may contribute to distinguishing feeding habits between them. This could potentially allow both predators to survive on a small island with limited resources and challenging environmental conditions.

**Figure 8:**
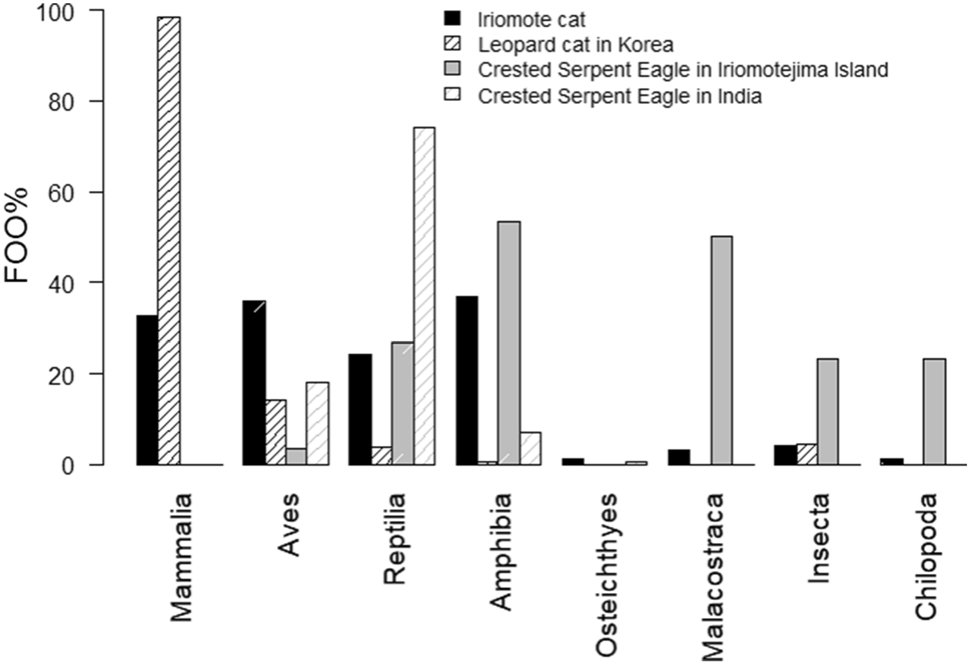
Comparison of frequency of occurrence (FOO) of each prey class between this study and previous studies which analyze the diet of the subspecies of the IRC and CSE, leopard cat in Korea^12^ and Crested Serpent Eagle in India^21^.

IRC and CSE subspecies in the Eurasian continent, that is, the leopard cat and the Crested Serpent Eagle, predominantly feed on rodents and snakes^11–14,20–22,26^. This study confirmed again that the diet of IRC and CSE are more varied than what has been previously found in other subspecies as shown in Fig. 8. Further diet analysis studies including other subspecies are needed to explore the evolutionary history of these animals along with their diet adaptations to Iriomote Island.

## Methods

### Study area

Iriomote Island has an area of approximately 284  km^2^ (24° 15–25ʹ N, 123° 40–55ʹ E), and is located in the southernmost part of the Ryukyu Archipelago, Japan (Fig. 2). The climate is subtropical, and dominated by *Castanopsis sieboldii* and *Quercus miyagii* that occupy most of the mountainous areas, with the highest elevation at 469 m. Human habitats are limited to lowland areas along the coast, and there are some cultivated areas around villages as well as swampy and mangrove forests along several rivers. The average temperature is 19.2 °C in winter (December to February) and 28.4 °C in summer (June to August), and the average amounts of rainfall are 483.0 mm and 597.2 mm in winter and summer, respectively (1991–2020, Japan Meteorological Agency).

### Faecal sampling

We collected faeces from IRC once a day during day light hours in the transects shown in Fig. 2 from November 2018 to February 2019 (winter) and June to September 2019 (summer). Faeces were identified by their shapes and smell. We also analyzed IRC faeces collected and preserved by the Monitoring Program of Okinawa District Forest Office of Forest Agency and Ministry of the Environment of Japan during 2015–2019 during the same month and the same north part of the island. CSE faeces on the road was collected once a day in the early morning in the transects shown in Fig. 2 at the same time as IRC. Fresh faeces of CSE were collected immediately after excretion observed from an adequate distance by the author (A.T.). Adherent uric acid mucus was manually removed to prevent PCR inhibition^41–43^. Additionally, we analyzed CSE faeces collected from individuals injured in traffic accidents and rescued by the Iriomote Wildlife Conservation Center of the Ministry of the Environment of Japan at the north part of the island. Faeces from both IRC and CSE were immediately preserved in a 100 mL wide-mouth bottle or 2 mL tube filled with 99% ethanol.

All faecal samples were derived from the north part of the Iriomote Island because sampling efforts are limited especially for CSE. The opportunity to collect their faeces is limited to a few hours in early in the morning when they appear in the location where A.T. could observe them and access their faeces. Detailed information about the sites where we collected the faeces could not be described here due to conservation concerns.

### DNA metabarcoding analysis

A total of 100–200 mg of small blocks of faeces were evenly partitioned from each sample and homogenized using a Micro Homogenizing System Micro Smash MS-100R (TOMY SEIKO, Tokyo, Japan) with sterilized 3.175 mm stemless beads. Each homogenate was then incubated at 50 °C for 120 min with the addition of 5.0 µL of proteinase *K* solution. Total DNA was extracted from each processed homogenate using a DNeasy PowerSoil Kit (Qiagen, Hilden, Germany), following the manufacturer’s protocol. Finally, 50 µL of total DNA solution was obtained from each sample.

First-round PCR was performed for each extracted DNA sample using universal primer sets to amplify partial mitochondrial genes derived from IRC and CSE prey species. Since the prey animals of these species are diverse, including vertebrates and invertebrates, we used four primer sets targeting different gene regions: Ecoprimer^44^ to amplify the vertebrate partial mitochondrial 12S ribosomal RNA (rRNA) gene, COI#1 (mlCOIintF and dgHCO2198)^45,46^ for metazoan partial mitochondrial cytochrome oxidase C subunit 1 (COI) genes, COI#2 (dgLCO1490 and COI-CFMRa)^47,48^ for the metazoan (especially insects) partial COI gene, and MtAnr for the anuran partial mitochondrial 16S rRNA gene (Supplementary Table S1). All PCR reactions were conducted under the same conditions: 10 µL mixtures were prepared for each PCR amplification, containing 1.0 µL of 10× Ex Taq Buffer (TaKaRa, Shiga, Japan), 0.80 µL of dNTP Mixture (TaKaRa), 0.10 µL of Ex Taq HS (TaKaRa), 0.60 µL (10 µM) of each primer, 1.0 µL of the extracted DNA solution, and 5.9 µL of nuclease-free water (Thermo Fisher Scientific, Waltham, MA, USA). The PCR conditions were as follows as Hebert et al.^49^: an initial denaturation at 94 °C for 1 min, followed by five cycles of 94 °C for 1 min, 45 °C for 1 min 30 s, and 72 °C for 1 min 30 s, followed by 35 cycles of 94 °C for 1 min, 50 °C for 1 min 30 s, and 72 °C for 1 min, and a final incubation at 72 °C for 5 min. All PCR reactions included negative controls.

Second-round PCR was conducted to add MiSeq (Illumina, San Diego, CA, USA) adaptor sequences and 8 bp index sequences to both ends of the first-round PCR products, as described by Miya et al.^50^. The PCR mixtures were prepared in a total volume of 10 µL, containing 1.0 µL of 10× Ex Taq Buffer (TaKaRa), 0.80 µL of dNTP Mixture (TaKaRa), 0.050 µL of Ex Taq HS (TaKaRa), 1.5 µL (10 µM) of each index primer, 1.0 µL of diluted 1st-round PCR products (30x) with nuclease-free water (Thermo Fisher Scientific), and 4.15 µL of nuclease-free water (Thermo Fisher Scientific). The PCR conditions were as follows: an initial denaturation at 98 °C for 1 min, followed by 12 cycles of 98 °C for 30 s, 65 °C for 30 s, 72 °C for 30 s, and final incubation at 72 °C for 5 min. We then pooled 3.0 µL of each 2nd-round PCR product for each respective primer set. The pooled samples were separated using 1.5% agarose gel electrophoresis using L03 Agarose (TaKaRa), and the DNA bands of the expected PCR target size for each primer set were dissected manually from the gel using a sterilized disposable scalpel. DNA was purified using a MinElute Gel Extraction Kit (Qiagen) following the manufacturer’s protocol. After purification using Agencourt AMPure XP (Beckman Coulter, Brea, California,USA) according to the standard protocol, the sample DNA concentration was quantified using the Qubit dsDNA HS assay kit and Qubit 4 Fluorometer (Thermo Fisher Scientific). DNA concentration was adjusted to 6.0 nM based on the expected amplicon size using nuclease-free water (Thermo Fisher Scientific). Finally, the prepared DNA library was subjected to 250 bp pair-end sequencing using MiSeq (Illumina), MiSeq Reagent Kits v2 (Illumina), and Micro flow cell (Illumina).

### Sequencing data analysis and species identification

From the obtained DNA sequence data, low quality 3' ends of the nucleotide sequences (Phred score < 10) were removed using the software DynamicTirm^51^. Paired-end reads were connected using the program FLASH^52^, and merged sequences less than 90 bp were discarded. Singleton sequences were removed using the program Uclust^53^, and the remaining sequences with more than two sequence counts were referred to as molecular operational taxonomic units (MOTUs). MOTUs were further filtered by their sequencing read numbers with a cut-off threshold of 0.01% of the total number of sequences in each sample. Sequence similarity-based Blast analysis^54^ was performed using the reference nucleotide database of NCBI (National Center for Biotechnology Information^55^). The blast hit record was adapted as a taxon of each MOTU with the following assignment methods: if the sequence similarity was (1) 99% or higher, the top-hit species was adopted, (2) 98% or higher but less than 99%, the top-hit genus was adopted, and (3) 98% or higher but less than 90%, the top-hit family was adopted^53^. If the multiple species were identified as the top-hit, the highest taxonomic rank below (1) genus, (2) order, and (3) family shared by all top-hit species were adopted^56^. Taxa and MOTUs were excluded if they did not inhabit Iriomote Island or if they were difficult to consider as prey items for IRC and CSE. These include IRC and CSE (the source of faeces), fungi, bacteria, humans, and insects less than 2 cm. The source species of each faecal sample was estimated based on the taxonomic assignment results of the Ecoprimer PCR products. MOTUs assigned to dogs and cattle were also excluded because they were considered environmental contaminants. We also excluded samples that did not contain any prey items from subsequent analyses as described in the results section.

### Statistical analysis

All statistical analyses were performed using *R* version 4.3.0 (*R* Core Team 2023). The quantitative data derived from the sequencing read number of each prey item were used to confirm the detectability of each primer set. The presence/absence data for each prey item were used for other analyses. The prey taxonomic richness was estimated with accumulation and extrapolation curves using ‘iNEXT’^57–59^. We utilized two metrics, frequency of occurrence (FOO) and weighted percentage of occurrence (wPOO)^60^ to conduct multivariate and statistical analyses. The results obtained from this study were examined by comparing the FOO values of each prey item with those obtained from a previous study that utilized morphological observations of faecal content^9^. Fisher’s exact test was used to examine the non-random relationships between the presence/absence ratio of each prey item for each predator to determine whether the differences between them were statistically significant. The weighted POO was not used in the comparative analysis because it was not suitable for interspecific comparisons^61^. To compare the diet composition of the two predators based on the Jaccard Index of the dissimilarity of the presence/absence data for prey items at the order level, we performed a non-metric multidimensional scaling (NMDS) analysis using the ‘vegan’ package^62^. The differentiation of the diet composition between these two species was confirmed by performing a permutational multivariate analysis of variance (PERMANOVA^63^) using the ‘adonis’ function. The ‘betadisper’ and ‘anova’ functions were also used to examine whether the differences were caused by different degrees of variation among the samples of IRC and CSE or by the different diet composition between these two species.

### Supplementary Information

Supplementary Information.

## Data Availability

The MiSeq-generated raw sequence reads from this study can be found in the DDBJ Sequence Read Archive (DRA) under the accession numbers DRA017514. The other data and computer code are available on Data Dryad (10.5061/dryad.x69p8czrf).

## References

[1] MacArthur RH, Wilson EO (1963). Equilibrium theory of insular zoogeography. Evolution.

[2] MacArthur RH, Wilson EO (1967). The Theory of Island Biogeography.

[3] Brown JH, Lomolino MV (1998). Biogeography.

[4] Okinawa Prefecture.* Outline of Yaeyama Region*. https://www.pref.okinawa.jp/site/norin/norin-yaeyama-nosui/keikaku/yaeyamanogaiyou.html (2016).

[5] Tamada T (2008). Molecular diversity and phylogeography of the asian leopard cat, felis bengalensis, inferred from mitochondrial and Y-chromosomal DNA sequences. Zool. Sci..

[6] Nakanishi N, Izawa M (2016). Importance of frogs in the diet of the Iriomote cat based on stomach content analysis. Mamm. Res..

[7] Sakaguchi N, Ono Y (1994). Seasonal change in the food habits of the Iriomote cat *Felis iriomotensis*. Ecol. Res..

[8] Watanabe, S.* Flexibility of Food Habit, Habitat Use, and Movement Pattern of the Iriomote Cat, Prionailurus bengalensis iriomotensis, as the Adaptation to the Insular Environment*. University of the Rykyus, Okinawa, Japan. https://u-ryukyu.repo.nii.ac.jp/records/2019242 (2004).

[9] Watanabe S, Ali M (2012). Ecological flexibility of the top predator in an island ecosystem—food habit of the Iriomote cat. Diversity of Ecosystems: Linking Structure And Function.

[10] Watanabe S, Nakanishi N, Izawa M (2003). Habitat and prey resource overlap between the Iriomote cat *Prionailurus iriomotensis* and introduced feral cat *Felis catus* based on assessment of scat content and distribution. Mamm. Study.

[11] Grassman LI, Tewes ME, Silvy NJ, Kreetiyutanont K (2005). Spatial organization and diet of the leopard cat (*Prionailurus bengalensis*) in north-central Thailand. J. Zool..

[12] Lee O, Lee S, Nam D-H, Lee HY (2014). Food habits of the leopard cat (*Prionailurus bengalensis euptilurus*) in Korea. Mamm. Study.

[13] Rajaratnam R, Sunquist M, Rajaratnam L, Ambu L (2007). Diet and habitat selection of the leopard cat (*Prionailurus bengalensis borneoensis*) in an agricultural landscape in Sabah, Malaysian Borneo. J. Trop. Ecol..

[14] Shehzad W (2012). Carnivore diet analysis based on next-generation sequencing: Application to the leopard cat (*Prionailurus bengalensis*) in Pakistan. Mol. Ecol..

[15] Watanabe S (2009). Factors affecting the distribution of the leopard cat *Prionailurus bengalensis* on East Asian islands. Mamm. Study.

[16] Watanabe S, Nakanishi N, Izawa M (2005). Seasonal abundance in the floor-dwelling frog fauna on Iriomote Island of the Ryukyu Archipelago, Japan. J. Trop. Ecol..

[17] DelHoyo J, Elliot A, Sargatal J (1994). Handbook of the Birds of the World, Vol. II. New World Vultures to Guineafowl.

[18] Mizutani, A., Nakamoto, J., Himura, S., Tanaka, S. & Kohno, H. Diet of the Crested Serpent Eagle *Spilornis cheela perplexus* observed in Iriomote and Ishigakijima, South Ryukyu Islands, Japan. *Study Rev. Iriomote Is. 2016, ORRC, Tokai Univ.* 25–43 (2017).

[19] Tokita H, Yoshino T, Onuma M, Kinjo T, Asakawa M (2014). Gastric contents of the Crested Serpent Eagle from Yaeyama Archipelago, Okinawa, Japan. Bird Res..

[20] Chou, T. C., Lee, P. F., & Chen, H. S. Breeding biology of the Crested Serpent-eagle *Spilornis cheela hoya* in Kenting National Park, Taiwan. *Raptors worldwide*, 557–568 (2004).

[21] Gokula V (2012). Breeding ecology of the crested serpent eagle *Spilornis cheela* (Latham, 1790) (Aves: Accipitriformes: Accipitridae) in Kolli hills, Tamil Nadu, India. TAPROBANICA J. Asian Biodiv.

[22] Pande S, Yosef R, Morelli F, Pawar R, Mone R (2018). Diet and habitat affinities in six raptor species in India. Avian Res..

[23] Schoener TW (1974). Resource partitioning in ecological communities. Science.

[24] Crooks KR, Van Vuren D (1995). Resource utilization by two insular endemic mammalian carnivores, the island fox and island spotted skunk. Oecologia.

[25] du Preez B, Purdon J, Trethowan P, Macdonald DW, Loveridge AJ (2017). Dietary niche differentiation facilitates coexistence of two large carnivores. J. Zool..

[26] Tatara M, Doi T (1994). Comparative analyses on food habits of Japanese marten, Siberian weasel and leopard cat in the Tsushima Islands, Japan. Ecol. Res..

[27] Ando H (2020). Methodological trends and perspectives of animal dietary studies by noninvasive fecal DNA metabarcoding. Environ. DNA.

[28] Monterroso P (2019). Feeding ecological knowledge: The underutilised power of faecal DNA approaches for carnivore diet analysis. Mamm. Rev..

[29] Shao X (2021). Generalist carnivores can be effective biodiversity samplers of terrestrial vertebrates. Front. Ecol. Environ..

[30] Shao X (2021). Prey partitioning and livestock consumption in the world’s richest large carnivore assemblage. Curr. Biol..

[31] Xiong M (2017). Molecular dietary analysis of two sympatric felids in the mountains of Southwest China biodiversity hotspot and conservation implications. Sci. Rep..

[32] Xiong M (2016). Molecular analysis of vertebrates and plants in scats of leopard cats (*Prionailurus bengalensis*) in southwest China. J. Mammal..

[33] Cleary GP, Corner LAL, O’Keeffe J, Marples NM (2011). Diet of the European badger (*Meles meles*) in the Republic of Ireland: A comparison of results from an analysis of stomach contents and rectal faeces. Mamm. Biol..

[34] Newton I (2004). Population limitation in migrants. Ibis.

[35] Tanaka M, Sato S (2019). The wintering habitats of the reed bunting (*Emberiza schoeniclus*) in Kochi City (Passeriformes: Emberizidae). Bull. Shikoku Inst. Nat. Hist..

[36] Schoch CL (2020). Database.

[37] Clarke LJ, Soubrier J, Weyrich LS, Cooper A (2014). Environmental metabarcodes for insects: In silico PCR reveals potential for taxonomic bias. Mol. Ecol. Resour..

[38] Deagle BE, Jarman SN, Coissac E, Pompanon F, Taberlet P (2014). DNA metabarcoding and the cytochrome c oxidase subunit I marker: Not a perfect match. Biol. Lett..

[39] Bookwalter J, Niyas AMM, Caballero-López B, Villari C, Claramunt-López B (2023). Fecal matters: implementing classical Coleoptera species lists with metabarcoding data from passerine bird feces. J. Insect Conserv..

[40] Walther BA, Ta-Ching C, Lee P-F (2014). Population density, home range, and habitat use of Crested Serpent-Eagles (*Spilornis cheela hoya*) in southern Taiwan: Using distance-based analysis and compositional analysis at different spatial cales. J. Raptor Res..

[41] Eriksson P, Mourkas E, González-Acuna D, Olsen B, Ellström P (2017). Evaluation and optimization of microbial DNA extraction from fecal samples of wild Antarctic bird species. Infect. Ecol. Epidemiol.

[42] Oehm J, Thalinger B, Eisenkölbl S, Traugott M (2017). Diet analysis in piscivorous birds: What can the addition of molecular tools offer?. Ecol. Evol..

[43] Oehm J, Juen A, Nagiller K, Neuhauser S, Traugott M (2011). Molecular scatology: How to improve prey DNA detection success in avian faeces?. Mol. Ecol. Resour..

[44] Riaz T (2011). EcoPrimers: Inference of new DNA barcode markers from whole genome sequence analysis. Nucleic Acids Res.

[45] Leray M (2013). A new versatile primer set targeting a short fragment of the mitochondrial COI region for metabarcoding metazoan diversity: Application for characterizing coral reef fish gut contents. Front. Zool..

[46] Meyer CP (2003). Molecular systematics of cowries (Gastropoda: Cypraeidae) and diversification patterns in the tropics. Biol. J. Linn. Soc..

[47] Geller J, Meyer C, Parker M, Hawk H (2013). Redesign of PCR primers for mitochondrial cytochrome c oxidase subunit I for marine invertebrates and application in all-taxa biotic surveys. Mol. Ecol. Resour..

[48] Jusino MA (2019). An improved method for utilizing high-throughput amplicon sequencing to determine the diets of insectivorous animals. Mol. Ecol. Resour..

[49] Hebert PDN, Cywinska A, Ball SL, DeWaard JR (2003). Biological identifications through DNA barcodes. Proc. R. Soc. B Biol. Sci..

[50] Miya M (2015). MiFish, a set of universal primers for metabarcoding environmental DNA from fishes: Detection of > 230 species from aquarium tanks and coral reefs in the subtropical Western North Pacific. Genome.

[51] Cox MP, Peterson DA, Biggs PJ (2010). SolexaQA: At-a-glance quality assessment of Illumina second-generation sequencing data. BMC Bioinform..

[52] Magoč T, Salzberg SL (2011). FLASH: Fast length adjustment of short reads to improve genome assemblies. Bioinformatics.

[53] Edgar RC (2010). Search and clustering orders of magnitude faster than BLAST. Bioinformatics.

[54] Camacho C (2009). BLAST+: Architecture and applications. BMC Bioinform..

[55] NCBI Resource Coordinators (2018). Database resources of the national center for biotechnology information. Nucleic Acids Res..

[56] Pereira A, Xavier R, Perera A, Salvi D, Harris DJ (2019). DNA metabarcoding to assess diet partitioning and feeding strategies in generalist vertebrate predators: A case study on three syntopic lacertid lizards from Morocco. Biol. J. Linn. Soc..

[57] Chao A (2014). Rarefaction and extrapolation with Hill numbers: A framework for sampling and estimation in species diversity studies. Ecol. Monogr..

[58] Hacker CE (2021). Use of DNA metabarcoding of bird pellets in understanding raptor diet on the Qinghai–Tibetan Plateau of China. Avian Res.

[59] Hsieh TC, Ma KH, Chao A (2016). iNEXT: an R package for rarefaction and extrapolation of species diversity (Hill numbers). Methods Ecol. Evol..

[60] Deagle BE (2019). Counting with DNA in metabarcoding studies: How should we convert sequence reads to dietary data?. Mol. Ecol..

[61] Wright BE (2010). Use of chi-square tests to analyze scat-derived diet composition data. Mar. Mamm. Sci..

[62] Oksanen J (2007). The vegan package. Commun. Ecol. Package.

[63] Anderson MJ, Kenett RS, Longford NT, Piegorsch WW, Ruggeri F (2017). Permutational multivariate analysis of variance (PERMANOVA). Wiley StatsRef: Statistics Reference Online.

