# Supplementary material for: Seasonal diet partition among top predators of a small island, Iriomote Island in the Ryukyu Archipelago, Japan
Source: Sci Rep. 2024 Apr 2;14:7727. doi: 10.1038/s41598-024-58204-6 (PMC10987585; doi:10.1038/s41598-024-58204-6)
Supplement: Supplementary file 1 — Supplementary Information. [file 41598_2024_58204_MOESM1_ESM.pdf]

Seasonal Diet Partition among Top Predators of a Small Island, Iriomote Island in the Ryukyu Archipelago, Japan

Alisa Tobe[1*], Yukuto Sato[2], Nakatada Wachi[3], Nozomi Nakanishi[4] and Masako Izawa[4*]

1. Faculty of Science, University of the Ryukyus, Nishihara, Okinawa, Japan (Present address: Wildlife Research Center, Kyoto University, Kyoto, Japan)
2. Center for Strategic Research Project, Organization for Research Promotion, University of the Ryukyus, Nishihara, Okinawa, Japan (Present address: Research Laboratory Center, Faculty of Medicine, University of the Ryukyus, Nishihara, Okinawa, Japan)
3. Center for Strategic Research Project, Organization for Research Promotion, University of the Ryukyus, Nishihara, Okinawa, Japan (Present address: Iriomote Station, Tropical Biosphere Research Center, University of the Ryukyus, Taketomi, Okinawa, Japa
4. Faculty of Science, University of the Ryukyus, Nishihara, Okinawa, Japan (Present address: Kitakyushu Museum of Natural History and Human History, Kitakyushu, Fukuoka, Japan)

Authors1 and 2 contributed equally to this work.

* Corresponding Authors.

Table S1. Detailed information on each primer set used in first-round PCR.

Primer set	Primer	Target gene	Target taxon	Primer sequences (5'-3')	Reference
Ecoprimer	12SV5-Fm	mtDNA 12S rRNA	vertebrate	TTAGATACCYYACTATGC	(Riaz et al. 2011, modified by this study)
	12SV5-R	mtDNA 12S rRNA		TAGAACAGGCTCCTCTAG	
COI#1	m1COIintF	mtDNA COI	metazoan	GGWACWGGWTGAACWGTWTAYCCYCC	(Leray et al. 2013, Mayer 2003)
	dgHCO2198	mtDNA COI		TAAACTTCAGGGTGACCAAARAAYCA	
COI#2	dgLCO1490	mtDNA COI	metazoan (insects)	GGTCAACAAATCATAAAGAYATYGG	(Geller et al. 2013, Jusino et al. 2017)
	COI-CFMRa	mtDNA COI		GGWACTAATCAATTTCCAAATCC	
MtAnr	MiT_Anr_F	mtDNA 16S rRNA	toads and frogs	CGGCGTWAAGCGTGATTAAAG	(This study)
	MiT_Anr_R	mtDNA 16S rRNA		GYCAARTCCTTTGGGTTTTAAGC	

Table S2. Frequency of occurrence (FOO%) in each prey item detected from each predator faeces. In summer, 16 items were identified from IRC faeces and 15 items from CSE faeces. In winter, 37 items were identified from IRC faeces and 14 items from CSE faeces. *P* values which are colored in red are $p < 0.05$ and in yellow are $p < 0.01$ (calculated by Fisher's exact test).

				Summer			Winter			*p* (between seasons)	
Class	Order	Family	Species	IRC (*N* =31)	CSE (*N* =21)	*p*	IRC (*N* =64)	CSE (*N*=9)	*p*	IRC	CSE
Mammalia	Cetartiodactyla	Suidae	*Sus scrofa*	0.00	0.00	N	4.69	0.00	1.000	0.549	N
Mammalia	Chiroptera	Hipposideridae	*Hipposideros turpis*	3.23	0.00	1.000	0.00	0.00	N	0.326	N
Mammalia	Chiroptera	Pteropodidae	*Pteropus dasymallus yayeyamae*	0.00	0.00	N	3.13	0.00	1.000	1.000	N
Mammalia	Eulipotyphla	Soricidae	*Suncus murinus*	3.23	0.00	1.000	0.00	0.00	N	0.326	N
Mammalia	Rodentia	Muridae	*Rattus rattus*	9.68	0.00	0.264	32.81	0.00	0.052	0.022	N
Aves	Columbiformes	Columbidae	*Chalcophaps indica*	3.23	0.00	1.000	0.00	0.00	N	0.326	N
Aves	Columbiformes	Columbidae	*Columba livia*	0.00	0.00	N	0.00	11.11	0.123	N	0.300
Aves	Columbiformes	Columbidae	*Streptopelia orientalis stimpsoni*	0.00	0.00	N	1.56	11.11	0.233	1.000	0.300
Aves	Columbiformes	Columbidae	*Treron formosae medioximus*	3.23	0.00	1.000	1.56	0.00	1.000	0.549	N
Aves	Galliformes	Phasianidae	*Gallus gallus*	0.00	0.00	N	6.25	0.00	1.000	0.300	N
Aves	Gruiformes	Rallidae	*Amaurornis phoenicurus*	3.23	0.00	1.000	7.81	0.00	1.000	0.660	N
Aves	Gruiformes	Rallidae	*Fulica atra*	0.00	0.00	N	1.56	0.00	1.000	1.000	N
Aves	Gruiformes	Rallidae	*Gallinula chloropus*	0.00	0.00	N	1.56	0.00	1.000	1.000	N
Aves	Gruiformes	Rallidae	*Rallina eurizonoides*	12.90	0.00	0.138	6.25	0.00	1.000	0.431	N
Aves	Passeriformes	Corvidae	*Corvus macrothynchos osai*	0.00	0.00	N	3.13	0.00	1.000	1.000	N
Aves	Passeriformes	Muscicapidae	Muscicapidae *sp.*	0.00	0.00	N	1.56	0.00	1.000	1.000	N
Aves	Passeriformes	Pycnonotidae	*Hypsipetes amaurotis stejnegeri*	3.23	0.00	1.000	0.00	0.00	N	0.326	N
Aves	Passeriformes	Turdidae	*Turdus pallidus*	0.00	0.00	N	1.56	0.00	1.000	1.000	N
Aves	Passeriformes	Turdidae	*Turdus sp.*	0.00	0.00	N	9.38	0.00	1.000	0.173	N
Aves	Passeriformes	Zosteropidae	*Zosterops japonicus loochooensis*	0.00	0.00	N	1.56	0.00	1.000	1.000	N
Aves	Passeriformes		Passeriformes *sp.*	3.23	0.00	1.000	1.56	0.00	1.000	0.549	N
Aves	Pelecaniformes	Ardeidae	*Ardea intermedia*	0.00	0.00	N	1.56	0.00	1.000	1.000	N
Aves	Pelecaniformes	Ardeidae	*Egretta sacra*	0.00	0.00	N	1.56	0.00	1.000	1.000	N
Aves	Pelecaniformes	Ardeidae	*Nycticorax nycticorax*	0.00	0.00	N	1.56	0.00	1.000	1.000	N
Aves	Strigiformes	Strigidae	*Otus elegans*	0.00	0.00	N	1.56	0.00	1.000	1.000	N
Reptilia	Squamata	Colubridae	*Cyclophiops herminae*	3.23	4.76	1.000	0.00	0.00	N	0.326	N
Reptilia	Squamata	Colubridae	*Dinodon rufozonatum walli*	0.00	0.00	N	0.00	11.11	0.123	N	0.300
Reptilia	Squamata	Colubridae	*Elaphe taeniura schmackeri*	9.68	0.00	0.264	0.00	0.00	N	0.032	1.000
Reptilia	Squamata	Colubridae	*Hebius ishigakiensis*	0.00	4.76	0.404	3.13	0.00	1.000	1.000	1.000
Reptilia	Squamata	Colubridae	*Lycodon ruhstrati multifasciatus*	0.00	0.00	N	1.56	0.00	1.000	1.000	N
Reptilia	Squamata	Elapidae	*Laticauda colubrina*	0.00	0.00	N	1.56	0.00	1.000	1.000	N
Reptilia	Squamata	Gekkonidae	*Gekko hokouensis*	0.00	4.76	0.404	0.00	11.11	0.123	N	0.517
Reptilia	Squamata	Lacertidae	*Takydromus dorsalis*	6.45	0.00	0.509	0.00	0.00	N	0.104	N
Reptilia	Squamata	Scincidae	*Plestiodon sp.*	48.39	14.29	0.017	1.56	0.00	1.000	<0.01	0.535
Reptilia	Squamata	Scincidae	*Plestiodon stimpsonii*	0.00	4.76	0.404	0.00	0.00	N	N	1.000
Reptilia	Squamata	Scincidae	*Scincella boettgeri*	0.00	0.00	N	0.00	33.33	<0.01	N	0.021
Amphibia	Anura	Dicroglossidae	*Fejervarya sakishimensis*	25.81	66.67	<0.01	14.06	0.00	0.590	0.252	<0.01

Table S2 (continued).

Class	Order	Family	Species	Summer IRC (*N* =31)	Summer CSE (*N* =21)	Summer *p*	Winter IRC (*N* =64)	Winter CSE (*N*=9)	Winter *p*	*p* (between seasons) IRC	*p* (between seasons) CSE
Amphibia	Anura	Microhylidae	*Microhyla kuramotoi*	0.00	9.52	0.158	9.38	22.22	0.255	0.173	0.563
Amphibia	Anura	Ranidae	*Nidirana okinavana*	0.00	0.00	N	1.56	0.00	1.000	1.000	N
Amphibia	Anura	Ranidae	*Odorrana supranarina*	3.23	9.52	0.558	21.88	0.00	0.192	0.018	1.000
Amphibia	Anura	Ranidae	*Odorrana utsunomiyaorum*	0.00	0.00	N	1.56	0.00	1.000	1.000	N
Amphibia	Anura	Ranidae	Ranidae *sp.*	0.00	4.76	0.404	1.56	0.00	1.000	1.000	1.000
Amphibia	Anura	Rhacophoridae	*Buergeria choui*	0.00	9.52	0.158	3.13	0.00	1.000	1.000	1.000
Amphibia	Anura	Rhacophoridae	*Rhacophorus owstoni*	0.00	0.00	N	10.94	11.11	1.000	0.092	0.300
Osteichthyes	Perciformes	Oxudercidae	*Periophthalmus argentilineatus*	0.00	0.00	N	1.56	0.00	1.000	1.000	N
Malacostraca	Decapoda	Gecarcinidae	*Discoplax hirtipes*	0.00	14.29	0.060	0.00	11.11	0.123	N	1.000
Malacostraca	Decapoda	Sesarmidae	*Chiromantes dehaani*	0.00	4.76	0.404	0.00	0.00	N	N	1.000
Malacostraca	Decapoda	Sesarmidae	*Sesarmops intermedium*	0.00	52.38	<0.01	4.69	22.22	0.112	0.549	0.229
Insecta	Hemiptera	Cicadidae	*Cryptotympana facialis*	3.23	23.81	0.034	1.56	0.00	1.000	0.549	0.286
Insecta	Lepidoptera	Sphingidae	*Hippotion celerio*	0.00	0.00	N	0.00	11.11	0.123	N	0.300
Insecta	Lepidoptera	Sphingidae	*Psilogramma menephron*	0.00	0.00	N	0.00	11.11	0.123	N	0.300
Insecta	Orthoptera	Tettigoniidae	*Mecopoda elongata*	0.00	0.00	N	0.00	11.11	0.123	N	0.300
Insecta	Orthoptera		Orthoptera *sp.*	0.00	0.00	N	3.13	0.00	1.000	1.000	N
Chilopoda	Scolopendromorpha	Scolopendridae	Scolopendridae *sp.*	0.00	19.05	0.022	1.56	22.22	0.038	1.000	1.000
Chilopoda	Scolopendromorpha	Scolopocryptopidae	Scolopocryptopidae *sp.*	0.00	0.00	N	0.00	11.11	0.123	N	0.300

Table S3. Potential prey Reptilia and Amphibia for IRC and CSE. *Detected by IRC in this study. **Detected by CSE in this study. ***Detected by both IRC and CSE in this study.

Class	Order	Family	*Species*
Reptilia	Testudines	Geoemydidae	*Mauremys mutica kami*
			Cuora flavomarginata evelynae
	Squamata	Gekkonidae	*Gekko hokouensis***
			Lepidodactylus lugubris
			Hemidactylus frenatus
			Hemiphyllodactylus typus
		Agamidae	*Japalura polygonata ishigakiensis*
		Scincidae	*Plestiodon kishinouyei*
			*Plestiodon stimpsonii***
			*Scincella boettgeri***
		Lacertidae	*Takydromus dorsalis**
		Typhlopidae	*Ramphotyphlops braminus*
		Pareatidae	*Pareas iwasakii*
		Colubridae	*Elaphe taeniura schmackeri**
			*Lycodon ruhstrati multifasciatus**
			*Dinodon rufozonatum walli***
			*Hebius ishigakiensis****
			*Cyclophiops herminae****
		Viperidae	*Protobothrops elegans*
		Elapidae	*Sinomicrurus macclellandi iwasakii*
			*Laticauda colubrina**
			Laticauda semifasciata
Amphibia	Anura	Ranidae	*Odorrana supranarina****
			*Odorrana utsunomiyaorum**
			*Nidirana okinavana**
			*Fejervarya sakishimensis****
		Rhacophoridae	*Rhacophorus owstoni****
			Kurixalus eiffingeri
			*Buergeria choui***
		Rhacophoridae	*Microhyla kuramotoi****

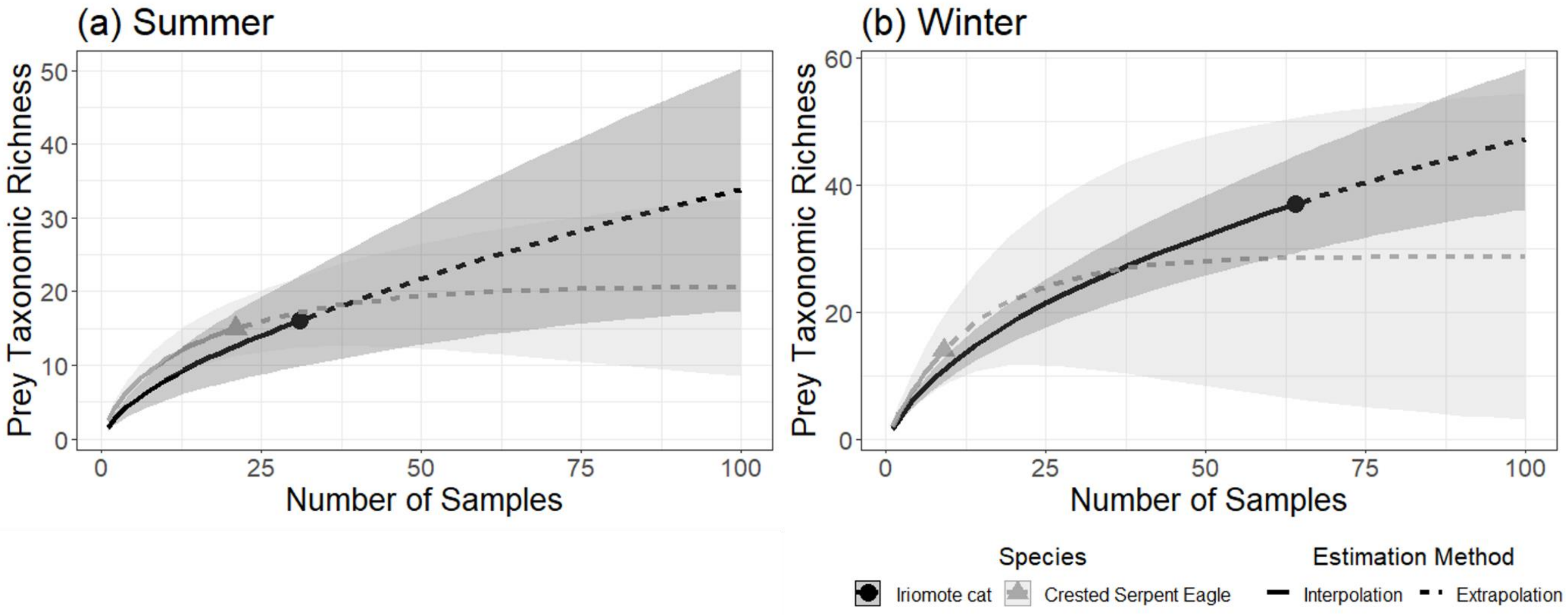

Figure S1. Species accumulation and extrapolation curve of prey taxonomic richness by number of samples (a) in summer and (b) in winter. Shaded area is 95% confidence interval.
